# RECAP reveals the true statistical significance of ChIP-seq peak calls

**DOI:** 10.1093/bioinformatics/btz150

**Published:** 2019-03-01

**Authors:** Justin G Chitpin, Aseel Awdeh, Theodore J Perkins

**Affiliations:** 1 Translational and Molecular Medicine Program, University of Ottawa, Ottawa, ON K1H8M5, Canada; 2 Regenerative Medicine Program, Ottawa Hospital Research Institute, Ottawa, ON K1H8L6, Canada; 3 School of Electrical Engineering and Computer Science, University of Ottawa, Ottawa, ON K1N6N5, Canada; 4 Department of Biochemistry, Microbiology and Immunology, University of Ottawa, Ottawa, ON K1H8M5, Canada

## Abstract

**Motivation:**

Chromatin Immunopreciptation (ChIP)-seq is used extensively to identify sites of transcription factor binding or regions of epigenetic modifications to the genome. A key step in ChIP-seq analysis is peak calling, where genomic regions enriched for ChIP versus control reads are identified. Many programs have been designed to solve this task, but nearly all fall into the statistical trap of using the data twice—once to determine candidate enriched regions, and again to assess enrichment by classical statistical hypothesis testing. This double use of the data invalidates the statistical significance assigned to enriched regions, thus the true significance or reliability of peak calls remains unknown.

**Results:**

Using simulated and real ChIP-seq data, we show that three well-known peak callers, MACS, SICER and diffReps, output biased *P*-values and false discovery rate estimates that can be many orders of magnitude too optimistic. We propose a wrapper algorithm, RECAP, that uses resampling of ChIP-seq and control data to estimate a monotone transform correcting for biases built into peak calling algorithms. When applied to null hypothesis data, where there is no enrichment between ChIP-seq and control, *P*-values recalibrated by RECAP are approximately uniformly distributed. On data where there is genuine enrichment, RECAP *P*-values give a better estimate of the true statistical significance of candidate peaks and better false discovery rate estimates, which correlate better with empirical reproducibility. RECAP is a powerful new tool for assessing the true statistical significance of ChIP-seq peak calls.

**Availability and implementation:**

The RECAP software is available through www.perkinslab.ca or on github at https://github.com/theodorejperkins/RECAP.

**Supplementary information:**

Supplementary data are available at *Bioinformatics* online.

## 1 Introduction

Chromatin Immunopreciptation (ChIP) followed by high-throughput sequencing, or ChIP-seq, has become a central approach to mapping transcription factor-DNA binding sites and studying the epigenome (Furey, 2012). ChIP-seq is the primary technique employed by a number of highly successful large-scale genomics projects, including ENCODE (Consortium *et al.*, 2012), modENCODE (Roy *et al.*, 2010), National Institutes of Health Roadmap Epigenomics Project (Kundaje *et al.*, 2015) and the International Human Epigenome Consortium (Stunnenberg *et al.*, 2016). Collectively, these projects have generated over 10 000 ChIP-seq datasets at a cost of 10 or 100 s of millions of dollars, while other smaller-scale projects have generated many more. Many biological inferences are based on these datasets, including DNA binding motifs of transcription factors (Mathelier *et al.*, 2014), regulatory elements and networks (Cheng *et al.*, 2014; Gerstein *et al.*, 2012; Griffon *et al.*, 2015), and possible connections to disease (Siggens and Ekwall, 2014). Thus, understanding exactly how much information we can or should extract from such data is a question of paramount importance.

Bioinformatics analysis of ChIP-seq data is a multi-stage process (Landt *et al.*, 2012), with the end goal of identifying genomic regions of possible transcription factor-DNA binding, histone positions, chromatin marks etc. There are numerous algorithms for identifying ChIP-seq enriched regions, or peak calling (e.g. Bardet *et al.*, 2013; Fejes *et al.*, 2008; Feng *et al.*, 2011b; Rashid *et al.*, 2011; Shen *et al.*, 2013; Spyrou *et al.*, 2009; Tuteja *et al.*, 2009; Valouev *et al.*, 2008; Xing *et al.*, 2012; Zang *et al.*, 2009; Zhang *et al.*, 2008). Because ChIP-seq data are noisy, virtually all peak calling algorithms output peaks with associated *P*-values. These *P*-values are useful for ranking peaks in decreasing order of confidence, and estimating false discovery rates (FDRs) at different significance thresholds. But how well can we trust the *P*-values produced by peak callers?

For our study, we chose to focus on three peak callers: MACS (version 2.1.1.20160309) (Feng *et al.*, 2011a, 2012; Zhang *et al.*, 2008), SICER (version 1.1) (Xu *et al.*, 2014; Zang *et al.*, 2009) and diffReps (version 1.55.6) (Shen *et al.*, 2013). We chose MACS because it is, at present, the most highly cited peak caller, and it is used by the ENCODE and modENCODE consortia for analysis of their data. SICER is another widely used and highly cited algorithm, but one designed more for the detection of the broad, regional enrichment characteristic of certain chromatin marks. This suits some of our experiments below, although MACS is also able to detect such regions, particularly when used in ‘broad peak’ mode. diffReps is designed to solve the differential enrichment problem—the comparison of two ChIP-seqs instead of a ChIP-seq and a control—which again comes up in some of our experiments.

Although these approaches to peak calling differ in a number of ways, all three (and many others from the list cited above) follow a common two-stage pattern: First, candidate peaks are identified by analyzing the ChIP-seq data, and second, those candidate peaks are evaluated for significance by comparing ChIP-seq data with some kind of control data. In the case of differential enriched region detection, two ChIP-seqs may be compared with each other by a similar process (Shen *et al.*, 2013). The problem with this design, as already pointed out by Lun and Smyth (2014), is that it commits the statistical sin of using the data twice. The ChIP-seq data are used to construct hypotheses to test, the candidate peaks, and then the same ChIP-seq data, along with control or other ChIP-seq data, is used to test those hypotheses by means of classical statistical hypothesis testing. In general, when the hypothesis and the test both depend on the same data, classical *P*-values cannot be trusted.

When peaks’ *P*-values are wrong, it creates a host of other problems. For one thing, we no longer have a good basis for choosing a *P*-value cut off for reporting results. Relatedly, we do not know how much we can trust any given peak, or even the set of peaks as a whole. If a peak has a *P*-value of 10^−10^, we might feel that is very likely to indicate true transcription factor binding or epigenetic modification. But if the peak caller is biased, so that the real statistical significance of such a peak is only 10^–10^, then perhaps we should not put much stock in it after all. FDR estimates, which are also reported by most peak callers, are virtually meaningless when based on *P*-values that are themselves incorrect. Another problem arises if we try to compare results from different peak callers. To make comparisons ‘fair’, we might restrict both peak callers to the same raw *P*-value (or FDR) cutoff. But if one algorithm has highly biased *P*-values and the other does not, then this comparison will hardly be fair. Finally, downstream analyses such as motif identification or regulatory network construction (Cheng *et al.*, 2014; Gerstein *et al.*, 2012; Griffon *et al.*, 2015; Mathelier *et al.*, 2014; Siggens and Ekwall, 2014) may be error-prone if we do not know which peaks are truly significant. This can be true even for rank-based downstream analyses such as the Irreproducible Discovery Rate (Li *et al.*, 2011) or motif discovery methods (Grau *et al.*, 2013; Kulakovskiy *et al.*, 2010). Although ranking peaks by *P*-value decreases the importance of the exact *P*-values themselves, the issue of where to cut off the list of candidate peaks remains and can influence results.

One approach to unbiased peak calling would be to develop a new peak calling approach from scratch, in a way that avoids double use of the data. However, as there are already many programs available that are satisfying in terms of identifying and ranking candidate peaks, with only their significance in question, we chose a different approach. We asked whether the *P*-values of peaks generated by these programs could be recalibrated to correct their bias. Happily, we found this to be largely possible through the new RECAP method that we introduce. RECAP stands both for the goal or our approach, recalibrating *P*-values, and the method by which it is done, resampling the read data and calling peaks again. RECAP is a wrapper algorithm that is compatible with almost any peak caller, and in particular MACS, SICER and diffReps, for which we provide wrapping scripts. RECAP pools and then resamples from the ChIP-seq and control data, approximating a null hypothesis scenario of no genuine difference between ChIP-seq and control. It then applies the peak caller to the resampled data, to estimate the distribution of *P*-values under that null hypothesis. It uses an estimated cumulative distribution function (CDF) of the null *P*-values to adjust the *P*-values produced by the peak caller on the original data. We show that on a variety of different types of simulated null hypothesis ChIP-seq data, where there is no actual enrichment, RECAP-recalibrated *P*-values are approximately uniformly distributed between zero and one—as should be the case for well-calibrated statistical hypothesis testing. This gives a more intuitive way of choosing a significance cutoff for peak calling, and allows us to look at whether default cutoffs (such as the 10^−5^ raw *P*-value cutoff in MACS) are overly conservative or still too loose. FDR estimates based on recalibrated *P*-values are also more reliable, and in particular, we show that FDR *q*-values for peaks in ENCODE data track well the reproducibility of those peaks between biological replicates. In summary, RECAP allows for much more rigorous and rational analysis of the significance of enrichment in ChIP-seq data, while allowing researchers to continue using the peak calling algorithms they already prefer and have come to depend on.

## 2 Algorithm

Our RECAP approach to recalibrating peak calling *P*-values is based on empirically estimating an expected CDF for those *P*-values, under the null hypothesis that the ChIP-seq and control read datasets are drawn from the same distribution across the genome. That is, if we were to view each read as an i.i.d. sample where different positions on the genome would have different probabilities of being sampled, then we assume the sampling distribution of ChIP-seq and control are identical. Some work (Boyle *et al.*, 2008; Ramachandran and Perkins, 2013) has explicitly attempted to estimate such distributions, but we will use a simpler mechanism for our *P*-value recalibration. The RECAP algorithm is summarized below.

### 2.1 The RECAP algorithm

- **Input:** Two mapped read datasets *T* (treatment or ChIP-seq) and *C* (control), peak calling algorithm *A*, and repeats number *R*
- **Call peaks:** Use algorithm *A* on datasets *T* and *C*, to generate peaks *P* with *P*-values $p=(p_{1},p_{2},\ldots ,p_{n})$
- **Model CDF of *P*-values under null hypothesis:**
  - Compute the union of all reads U=T∪C
  - For *i* = 1 to *R* do:
    - Randomly divide *U* into mock treatment *T_i_* and control *C_i_*, with the same numbers of reads as *T* and *C*, respectively
    - Call peaks using *A* on datasets *T_i_* and *C_i_* generating peaks with *P*-values $p^{i}=(p_{1}^{i},p_{2}^{i},\ldots ,p_{n_{i}}^{i})$
  - Combine all resampled *P*-values, along with an extra value *P* = 0, into a single sorted list of unique values $\left(p_{\left(1\right)},p_{\left(2\right)},\ldots ,p_{\left(m\right)}\right)$ along with the number of times each value occurred $\left(N_{1},N_{2},\ldots ,N_{m}\right)$.
  - Estimate the null *P*-value CDF
    $$F(x)=\{\begin{array}{cc} \left(\sum_{j=1}^{i}N_{j}\right)/\left(\sum_{j=1}^{m}N_{j}\right) & \;\text{if}\;x=p_{\left(i\right)}\\ \frac{\left(\sum_{j=1}^{i}N_{j}\right)+N_{i+1}\left(\frac{x-p_{\left(i\right)}}{p_{\left(i+1\right)}-p_{\left(i\right)}}\right)}{\sum_{j=1}^{m}N_{j}} & \;\text{if}\;p_{\left(i\right)}<x<p_{\left(i+1\right)}\\ 1 & \;\text{if}\;x>p_{\left(m\right)} \end{array}$$
- **Output:** Original peak set *P* with recalibrated *P*-values
  $$p\prime =(F(p_{1}),F(p_{2}),\ldots ,F(p_{n}))$$

The intuition behind the algorithm is that if the null hypothesis holds, we can simulate new-but-similar treatment and control datasets by resampling from the combined reads of the original treatment and control. If we do that one or more times, and call peaks each time, we can estimate an average-case distribution of *P*-values for similarly distributed data. We use a linearly interpolated empirical CDF estimate to capture that distributional information, and to correct the original *P*-values. The advantage of the linearly interpolated CDF compared with the standard piecewise-constant empirical CDF estimate ($F(x)=\left(\sum_{j=1}^{i}N_{j}\right)/\left(\sum_{j=1}^{m}N_{j}\right)$, where $p_{\left(i\right)}\leq x<p_{\left(i+1\right)}$) is that the former is a strictly monotone mapping for $x\in [0,p_{\left(m\right)}]$. Thus, recalibrated *P*-values will have the same ranking as the original, raw *P*-values, with the possible exception of any raw *P*-values larger than $p_{\left(m\right)}$.

Implicitly, our approach makes the assumption that there is some null distribution of *P*-values to estimate. In principle, every resampling of the data might generate peaks with radically different *P*-values or produce no peaks at all. If resampled data generated no peaks at all, then of course it would be impossible to estimate a null *P*-value distribution. In preliminary testing of all three algorithms, we found that while the numbers of peaks called could vary considerably between different resamples (particularly for MACS), the distributions of *P*-values were largely the same. Moreover, the number of resamplings *R* had little effect on recalibrated *P*-values. If that were not true, then one might want to think more carefully about how to combine *P*-values from different resamplings.

Our approach also assumes that every peak in every resampling of the data is an i.i.d. sample from the null *P*-values distribution. Because peak calling relies in part on local read densities, nearby peaks have non-statistically independent *P*-values. However, because these dependencies typically do not span a large portion of the genome, we expect the independence assumption is reasonable.

## 3 Results and discussion

### 3.1 MACS, SICER and diffReps produce biased *P*-values

**Fig. 1.**
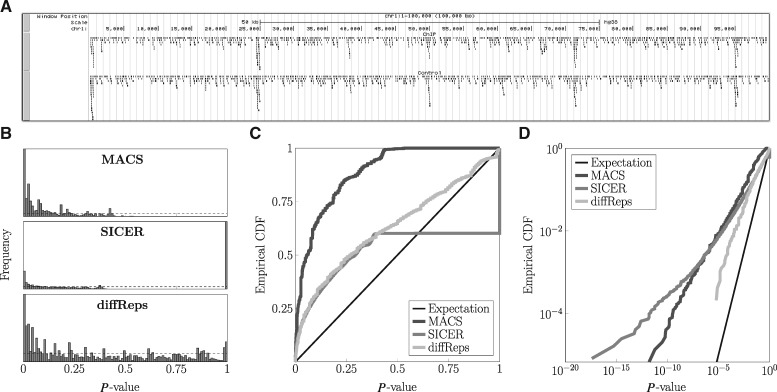
MACS, SICER and diffReps peak callers produce biased *P*-values. **(A)** Visualization of part of a simulated ChIP-seq read dataset, with 500 bp foreground regions every 20–25 kb, where read density is greater. Control data was generated similarly, with matching foreground regions, so a null hypothesis of no enrichment in ChIP-seq versus control is true for every possible genomic region. **(B)** Peaks called by the three algorithms have *P*-values that are not uniformly distributed between zero and one, as should be the case for this null hypothesis data if *P*-values were well calibrated. Empirical CDFs on linear **(C)** and log **(D)** axes also show the discrepancy from the uniform distribution

Before reporting on RECAP, we felt it was important to establish definitively whether different peak callers do, as suspected, produce peaks with biased *P*-values. As an initial test, we generated 10 simulated null hypothesis datasets. In each dataset, both ChIP-seq and control data comprise foreground regions and background regions. Foreground regions are 500 bp long and spread ∼20–25 kb apart along a set of chromosomes of the same number and sizes specified by the hg38 genome assembly (including both X and Y chromosomes). Foreground regions are the same for both ChIP-seq and control. Each ChIP-seq and control dataset had 30 882 698 reads—one per 100 bp of the genome on average. A 10% of the reads were placed uniformly randomly within the foreground regions, while the remainder was placed uniformly randomly within the background regions. Figure 1A shows a zoom-in on part of one of the randomly generated ChIP-seq datasets and its matching control. We chose these parameters for numbers of peaks, peak size and total reads to be broadly consistent with current ENCODE datasets.

We ran MACS, SICER and diffReps on these datasets, using default parameters with one exception. We set *P*-value or FDR cutoff thresholds at or as close as possible to 1.0, so that all candidate peaks, regardless of significance, would be reported. Figure 1B shows histograms of the *P*-values of the peaks produced by each program, for one of the 10 simulated ChIP-seq-control dataset pairs. Results for the other nine datasets were similar.

By near universal definition, a *P*-value is the chance of observing data as or more ‘extreme’ than some given data (Wasserman, 2013), under some null statistical model. As such, when applied to null-generated data, a well-calibrated method for calculating *P*-values should output *P*-values that are approximately uniformly distributed on [0, 1]. That all three peak callers’ *P*-value distributions are non-uniform is visually clear from Figure 1B, where the horizontal dashed lines indicate the uniform distribution, and from Figure 1C, where we plot the empirical CDFs of the *P*-values of the three programs. Well-calibrated *P*-values should have empirical CDF close to the thin black diagonal line. Although we will momentarily introduce a different statistic for quantifying deviation from uniformity, a simple KS-test shows that the three *P*-value distributions of the programs are statistically significantly different from the uniform distribution (P≈0 incalculably small for all three).

Figure 1D shows the same empirical CDFs, but plotted on log–log axes. This plot is informative because most *P*-values are close to zero, and it is difficult to see their distribution on linear axes. Again, this plot shows that all three algorithms produce *P*-values that are non-uniformly distributed, and in particular, optimistically biased compared with the expectation under a uniform distribution of *P*-values. But it is now much more clear that diffReps’s *P*-values are the closest to being uniformly distributed, whereas MACS’s and SICER’s *P*-value distributions are farther afield. The curve for SICER, in fact, grows worse as *P*-value get smaller; SICER seems particularly prone to outputting highly significant *P*-values. Motivated by this log–log plot of empirical CDFs, we propose a measure of deviation from uniformity. For a given set of *N P*-values, we let $N_{1}/N$ be the fraction of those *P*-values in the range $[0.1,1],\;N_{2}/N$ be the fraction in the range [0.01,0.1), and more generally $N_{i}/N$ be the fraction in the range $\left[10^{-i},10^{-i+1}\right)$. Then we quantify deviation from uniformity by the statistic: $D={\text{mean}}_{i:N_{i}>0}|{\;\text{log}\;}_{10}(N_{i}/N)-{\;\text{log}\;}_{10}(9\times 10^{-i})|$. In words, this is the absolute difference between the logarithm of the fraction of peaks that should be in a *P*-value bin and the logarithm of the fraction of peaks that actually are in the bin, averaged over the non-empty bins. If a set of *P*-values is uniformly distributed on [0,1], so that 90% of them fall in [0.1,1], 9% fall in [0.01,0.1) etc., then *D* evaluates to zero. Non-uniform distributions produce higher values of *D*. An advantage of this measure compared, for example, to the statistic used by the KS-test is that it pays equal attention to *P*-values at many different significance levels. In contrast, the KS-test looks at the maximum difference between the empirical CDF and the theoretical uniform CDF. For the SICER data, e.g. this maximum occurs at *P* = 1, where ∼40% of the peaks are. But the peaks with such high *P*-values are not of any biological interest, so it is undesirable for a performance metric to emphasize them to the exclusion of all else. For the present data, the deviations of the three algorithms’ *P*-value distributions evaluate to D≈2.8 for MACS, D≈6.1 for SICER and D≈0.9 for diffReps.

Although we will quantify bias and its removal more thoroughly in the next section, several important points remain regarding biases in the *P*-values produced by these programs. First, our results are not an artifact of the precise way the simulated null hypothesis ChIP-seq and control datasets were generated. For example, we also generated data with similar foreground regions but with 20% of reads in the foreground and 80% in the background. We also generated data with broad foreground regions of 4 kb containing 30% of the reads, leaving 70% for the background. For these datasets, we ran MACS in broad peak mode. In all cases, we continue to see deviation from uniformity in the *P*-value distributions (Supplementary Fig. S1A–C). Second, the amount of bias in these *P*-value distributions differs for the different types of data and for the different algorithms. This means that there is no universal correction that can be applied to the *P*-values, to bring them into line. Bias removal must operate in a way specific to the data being analyzed and to the program being used to call peaks.

Finally, it is important to note that evidence of bias can be seen in real data, not just simulated data. To show this, we turned to ChIP-seq data from the ENCODE consortium (Consortium *et al.*, 2012). We chose to analyze data from the five cell types with the most available datasets, namely K562, A549, GM12878, HepG2 and myocytes. For each cell type, we identified all experiments conducted by the same lab that included two replicate ChIP-seq experiments and two matching controls, and chose (arbitrarily) 10 replicate pairs for analysis (see Supplementary Table S1). In an attempt to approximate null hypothesis-like conditions, but using real data, we called peaks on each ChIP-seq dataset using its ChIP-seq replicate as control. The resulting *P*-value CDFs for all three algorithms are shown in Supplementary Figure S1D–R. As with our simulated data, we see all the CDFs are optimistically biased, in some cases returning dramatic *P*-values exceeding $10^{-300}$. Thus, *P*-value bias is not just an artificial theoretical concern, but a genuine concern that is observable and should be expected in the analysis of real data.

### 3.2 RECAP removes bias from peak caller *P*-values

**Fig. 2.**
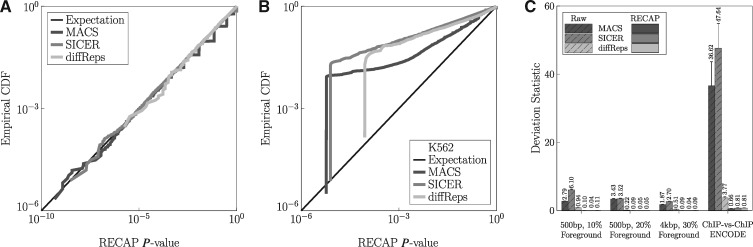
RECAP recalibrates peak callers' *P*-values to a near-uniform distribution. **(A)** Log-log plot of the empirical CDF of recalibrated *P*-values for MACS, SICER and diffReps, on the simulated, 10% foreground, null-hypothesis data. **(B)** Similar plot for a representative ChIP-seq replicate pair from ENCODE. **(C)** Reductions in deviation statistic, which measures difference from the uniform distribution, for the RECAP-recalibrated *P*-values for several types of simulated data (10 datasets each) and 50 matched pairs of ENCODE replicate ChIP-seq data (10 pairs for each of five cell types)

We tested RECAP’s ability to correct bias in peak *P*-values on a variety of simulated and real null hypothesis datasets. Figure 2A shows the results for the same 10%-reads, 500 bp foreground region dataset used for Figure 1B–D. Comparing particularly Figure 2A with Figure 1D, we see that RECAP has very substantially removed the bias. The log–log plot of the *P*-value CDFs for all three algorithms is very close to the expectation line.

When applying RECAP to replicate pairs of ENCODE ChIP-seq data, we obtained a reduction but not elimination of bias. Figure 2B shows the log–log CDF plot for one of the real ChIP-seq pairs. We see that all three peak callers’ *P*-values remain above the expectation line, and thus are optimistically biased. However, we do not see recalibrated *P*-values on the order of $10^{-100}$ or even smaller. Indeed, because of the extra *P* = 0 *P*-value we inject into our resampled peak *P*-values (see Section 2), the smallest recalibrated *P*-value can be no smaller than 1/(z+1), where *z* is the total number of peaks in all resampled peak calls. This feature is responsible for the way the empirical CDFs of recalibrated *P*-values drop down near the expectation line on the left side. Without the extra *P* = 0 resampled *P*-value, even the linearly interpolated CDF can output extremely small recalibrated *P*-values, if they fall below any resampled *P*-values. The fact that bias is not completely eliminated could be the result of genuine differences between the two replicates, causing peaks to appear in one and not the other, or causing overall signal fidelity to be different. Indeed, visual inspection of read pileups suggests a better peak signal-to-noise ratio in one of the datasets.

Supplementary Figure S2A–O shows the log-log empirical CDFs of recalibrated *P*-values for all 10 ChIP-seq replicate pairs, for all five cell types. The general trend is that many datasets still show some optimistic *P*-value bias, while some show good calibration. Just a few appear over-corrected, with CDFs falling below the expectation line. Comparing to the raw *P*-value CDFs in Supplementary Figure S1D–R, we see that bias has been very substantially reduced, although MACS and diffReps *P*-values remain somewhat optimistically biased. A quantitative summary of bias before and after recalibration by RECAP is in Figure 2C and Supplementary Figure S3. As was apparent visually, in all cases, we see that *P*-value distribution bias, as quantified by our deviation statistic *D*, is very substantially reduced.

**Fig. 3.**
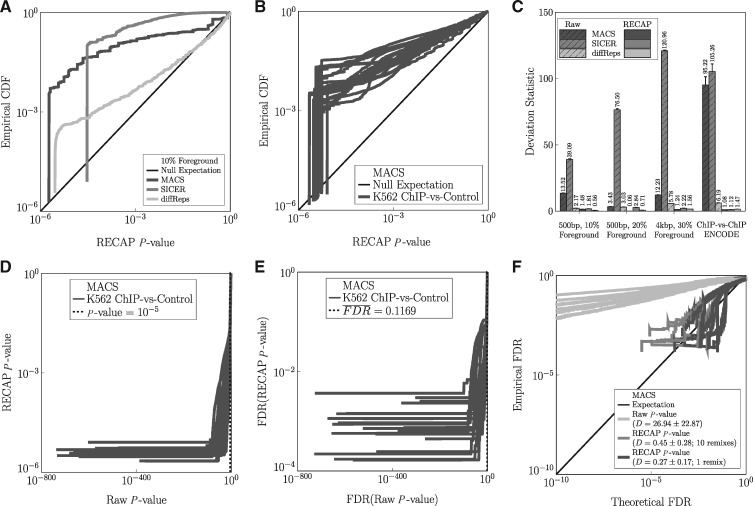
FDR and reproducibility analyses based on recalibrated *P*-values. **(A)** Empirical CDF of recalibrated *P*-values for each algorithm on simulated non-null data. **(B)** Empirical CDFs of MACS’s *P*-values on ENCODE datasets. **(C)** Deviation statistics before and after recalibration. **(D)** Raw versus recalibrated *P*-values for MACS on ENCODE data. **(E)** FDR estimates based on recalibrated versus raw *P*-values for MACS on ENCODE data. **(F)** Peak reproducibility rates versus FDR estimates based on raw or recalibrated *P*-values

### 3.3 Peak statistical significance and FDRs estimated by RECAP

When *P*-values are not well-calibrated, the true statistical significance of individual peaks is unknown, and FDR estimates based on those *P*-values cannot be trusted. Conversely, if *P*-values are well-calibrated, then FDR estimates based on those *P*-values should also be well-calibrated. To examine these assertions, we ran further tests on both simulated and real (ENCODE) data. For the simulated data, we used as treatment the same datasets described earlier, but we used as control an equal number of reads distributed uniformly randomly across the genome. As such, there are many genuinely enriched regions in each simulated ChIP-seq dataset. For the real data, we focused on the same 100 ENCODE ChIP-seq datasets mentioned in the previous section (5 cell types × 10 experiments × 2 ChIP-seq replicates per experiment). We ran all three peak callers on each of the 100 ChIP-seqs independently, using matched controls as specified by the ENCODE project website (www.encodeproject.org; see Supplementary Table S2). We ran RECAP with either 1 or 10 resamplings to recalibrate the *P*-values. Based on the recalibrated *P*-values, we then computed *q*-values based on the method of Benjamini and Hochberg (1995).

Figure 3A shows empirical CDFs of re-calibrated *P*-values for one typical simulated dataset with 10% of reads in 500 bp peaks. We observe that even after correction, the CDFs are significantly above the null expectation. This indicates genuine difference between treatment and control, which we know is correct for this data. Figure 3B shows similar behavior for MACS on the K562 ChIP-seq datasets (see Supplementary Fig. S4A–P for the analogous plots for the other peak callers and cell types). The *P*-value distributions shown in Figure 3B, where we call peaks on ChIP-seq versus control, are similar to the distributions shown in Supplementary Figure S2A, where we call peaks on ChIP-seq versus replicate ChIP-seq. However, the ChIP-seq versus control curves shows more deviation from the null hypothesis, as we summarize quantitatively in Figure 3C. Indeed, across all cell types and peak callers, recalibration lowers the deviation statistics. However, it does not lower them as much as when we call simulated or real ChIP-seqs against each other (compare with Fig. 2C). This indicates, as one would expect, that although replicate ChIP-seqs may have some differences, there are more differences between ChIP-seqs and controls, and more strongly genuinely enriched regions.

Beyond the deviation statistics, a point of central interest is how *P*-values are transformed by calibration when there are genuinely enriched regions. Figure 3D and Supplementary Figure S4S-AD plot recalibrated against raw *P*-values for MACS, SICER and diffReps. For MACS and SICER, peaks with phenomenal *P*-values like $10^{-200}$ have significance upon recalibration on the order of $10^{-3}$ to $10^{-6}$. Although many of these may still be significant, their level of significance is not nearly what one might have expected. For diffReps, we find that the *P*-values, while optimistic, are not nearly so biased on the ENCODE data, and are typically recalibrated by an order of magnitude or less. For MACS, the default raw *P*-value cutoff is $10^{-5}$. Across the 100 ENCODE ChIP-seqs, we found that the least significant peak (i.e. the one with largest *P*-value ≤ 10^ − 5^) had a recalibrated *P*-value consistently near 0.5 (mean 0.5669, SD 0.2335). If we apply the same raw *P*-value cutoff to SICER peaks, the least significant peaks have a recalibrated significance of 0.6072 ± 0.2595. For diffReps, however, these least significant peaks have better calibrated raw *P*-values of 0.0504 ± 0.1816.

Figure 3E and Supplementary Figure S4AE–AR show FDR estimates (*q*-values) based on recalibrated versus uncorrected *P*-values. Again, we see a great discord between the two, particular for MACS and SICER. For MACS e.g. uncorrected FDR estimates between $10^{-30}$ and $10^{-60}$—which would suggest no false positives at all in a typical set of peak calls—map to recalibrated FDR estimates between 10^–1^ and 10^−4^—suggesting a much higher level of false positives. If we look at the peaks up to an uncalibrated *q*-value threshold of 10^−5^, FDRs estimated based on our recalibrated *P*-values are 0.1000 ± 0.0904 for MACS, 0.5337±0.2501 for SICER, and 0.0649 ± 0.1933 for diffReps. Given that datasets such as these ENCODE ones typically have thousands or tens of thousands of peaks, the difference in uncalibrated and calibrated FDR estimates means the difference between essentially zero estimated false positive peaks and hundreds or even thousands of estimated false positive peaks.

Finally, we turn to the question of whether theoretical FDR estimates correlate to empirical FDRs. To do this for the ENCODE ChIP-seq replicate pairs, we designated a peak called in a ChIP-seq dataset as a true positive if it overlaps (by as little as one basepair) with a peak called in the replicate dataset. Otherwise, a peak is designated a false positive. In Figure 3F and Supplementary Figure S4AW, AZ, BC and BF, we plot empirical FDRs against FDR estimates based on either uncalibrated or recalibrated *P*-values. For MACS, theoretical FDRs based on uncorrected *P*-values are optimistic. For example, a theoretical FDR of $10^{-5}$, at which level there should be essentially no false positives, corresponds to an empirical FDR (i.e. reproducibility failure rate) of ∼10^−2^, suggesting hundreds of peaks would be false positives. However, we see that theoretical FDRs based on recalibrated *P*-values track fairly well the empirical FDR, as seen by the clustering of the curves around the ‘expectation’ line. Thus, for MACS, we suggest that FDR calculations based on recalibrated *P*-values can be trusted as a rough approximation of reproducibility, whereas FDRs based on uncalibrated *P*-values should not be taken at face value. For SICER the story is slightly more complicated. In Supplementary Figure S4AS, AU, AX, BA and BD we see (and the deviation statistic in Supplementary Fig. S5 confirms it) that FDR estimates based on recalibrated *P*-values more accurately reflect empiricial reproducibility. However, there is high variability across datasets, so that it is difficult to trust the results on any given dataset. For diffReps (Supplementary Fig. S4AT, AV, AY, BB and BE), the bias in *P*-values was already low. Nevertheless, we see a modest improvement in the accuracy of FDR estimates based on recalibrated *P*-values versus estimates based on the raw *P*-values.

## 4 Conclusions

In this article, we considered the statistical significance of ChIP-seq peak calls, on the grounds that ‘double use’ of the data by programs such as MACS, SICER and diffReps would likely lead to optimistic bias in *P*-values. Tests using simulated null hypothesis data, where there is no enrichment between treatment and control, confirmed this suspicion. We found similar results on replicate ChIP-seq pairs from the ENCODE project. We then described RECAP, a wrapper algorithm that can substantially reduce or eliminate *P*-value bias. It does so by using resampled data to estimate a null distribution of *P*-values, in a manner that is specific to a dataset and peak caller. Recalibrated *P*-values obtained for ENCODE data suggest that raw *P*-values can be overly significant by many orders of magnitude, and FDRs may be ∼100 times higher than previously estimated—although it remains clear that there are many genuinely enriched regions in these datasets.

Although RECAP is a complete system as it stands, there are a number of possible directions for further work. First, while we showed our recalibrated *P*-values are useful for more accurate FDR estimation, they should also be useful for local FDR estimation (Efron, 2007). Although global FDR analysis tells us how many false positives may be in a given set of returned results, local FDR analysis can tell us how likely any individual peak is to be a false positive. Local FDR analysis requires a scheme for estimating null and non-null *P*-value distributions, and the prior probability of true versus false peaks. Second, we note that our *P*-value recalibration scheme monotonically transforms raw *P*-values based on an empirical CDF. This means that if one peak is more significant than another by raw *P*-value, its recalibrated *P*-value will also be more significant. But plausibly, some kinds of peaks or some genomic regions are more likely to be false positives than others. Indeed, other ongoing work in ChIP-seq analysis aims at uncovering and removing local biases in ChIP-seq signals that can unduly influence peak calling (Hiranuma *et al.*, 2016, 2018; Ramachandran *et al.*, 2015). This suggests that peak-specific *P*-value corrections might be desirable, although it is unclear how this can best be done. Finally, although we have focused here on ChIP-seq peak calling, it is entirely reasonable to think that similar problems with *P*-value calibration may occur in other areas of high-throughput data analysis. For example, this may occur in DNA variant-calling, where complex conditions of uni- or bi-directional read coverage or other types of pre-filtering are sometimes applied before candidate variants are tested statistically. This double-usage of the data, to both select hypotheses for testing and to compute significance for those hypotheses, is a recipe for biased *P*-values. Perhaps in such cases, a similar read-resampling scheme could be used to calibrate *P*-values output by different variant callers.

## Funding

This work was supported in part by Discovery Grant [328154-2014] from the Natural Sciences and Engineering Research Council of Canada (NSERC).

*Conflict of Interest*: none declared.

## Supplementary Material

btz150_Supplementary_Information
