# Supplementary material for: RECAP reveals the true statistical significance of ChIP-seq peak calls
Source: Bioinformatics. 2019 Mar 1;35(19):3592–8. doi: 10.1093/bioinformatics/btz150 (PMC6761936; doi:10.1093/bioinformatics/btz150)
Supplement: btz150_Supplementary_Information [file btz150_supplementary_information.pdf]

Supplementary information for:

RECAP reveals the true statistical significance of ChIP-seq peak calls

Justin G. Chitpin, Aseel Awdeh, Theodore J. Perkins

Table S1: ENCODE identifiers for ChIP-seq replicate datasets

Replicate 1	Replicate 2	Lab	Transcription Factor	Cell Line
ENCFF263MBV	ENCFF860IRS	Michael Snyder	TARDBP	*Homo sapiens* K562
ENCFF141XAC	ENCFF995IZW	Michael Snyder	SKIL	*Homo sapiens* K562
ENCFF487SUP	ENCFF758LME	Michael Snyder	SP1	*Homo sapiens* K562
ENCFF254IUH	ENCFF167NVK	Michael Snyder	MTA2	*Homo sapiens* K562
ENCFF294UTM	ENCFF387BDW	Michael Snyder	SMARCE1	*Homo sapiens* K562
ENCFF619XNM	ENCFF518PQY	Michael Snyder	ATF7	*Homo sapiens* K562
ENCFF024NZQ	ENCFF168TBA	Michael Snyder	RAD51	*Homo sapiens* K562
ENCFF682OCC	ENCFF639CLN	Michael Snyder	MIER1	*Homo sapiens* K562
ENCFF149LTH	ENCFF873BRB	Michael Snyder	ZBTB40	*Homo sapiens* K562
ENCFF717OYW	ENCFF500YYZ	Michael Snyder	ZEB2	*Homo sapiens* K562
ENCFF073MBT	ENCFF280ZFT	Tim Reddy	CEBPB	*Homo sapiens* A549
ENCFF074CYV	ENCFF864XTU	Tim Reddy	EP300	*Homo sapiens* A549
ENCFF796UCB	ENCFF098QVB	Tim Reddy	BCL3	*Homo sapiens* A549
ENCFF179XAQ	ENCFF330XFU	Tim Reddy	JUNB	*Homo sapiens* A549
ENCFF217RBI	ENCFF791DRP	Tim Reddy	CEBPB	*Homo sapiens* A549
ENCFF347MNU	ENCFF417GPF	Tim Reddy	CEBPB	*Homo sapiens* A549
ENCFF599JTK	ENCFF389OFH	Tim Reddy	JUNB	*Homo sapiens* A549
ENCFF476XBN	ENCFF761UEZ	Tim Reddy	JUN	*Homo sapiens* A549
ENCFF504YVD	ENCFF595EIS	Tim Reddy	HES2	*Homo sapiens* A549
ENCFF181HLP	ENCFF870WJP	Tim Reddy	NR3C1	*Homo sapiens* A549
ENCFF575GRT	ENCFF177XDM	Richard Myers	TAF1	*Homo sapiens* GM12878
ENCFF725DLZ	ENCFF238NNG	Richard Myers	EP300	*Homo sapiens* GM12878
ENCFF399QIP	ENCFF802IZN	Richard Myers	BATF	*Homo sapiens* GM12878
ENCFF579PRC	ENCFF884LEJ	Richard Myers	RUNX3	*Homo sapiens* GM12878
ENCFF791EPM	ENCFF845MYC	Richard Myers	PBX3	*Homo sapiens* GM12878
ENCFF963FTZ	ENCFF179SRP	Richard Myers	TARDBP	*Homo sapiens* GM12878
ENCFF240MQI	ENCFF888PAI	Richard Myers	IRF4	*Homo sapiens* GM12878
ENCFF894EID	ENCFF569QEN	Richard Myers	REST	*Homo sapiens* GM12878
ENCFF207QTV	ENCFF983YCI	Richard Myers	NFATC1	*Homo sapiens* GM12878
ENCFF731ZNW	ENCFF263NOT	Richard Myers	SRF	*Homo sapiens* GM12878

Replicate 1	Replicate 2	Lab	Transcription Factor	Cell Line
ENCFF013XEG	ENCFF040PMB	Xiang-Dong Fu	TARDBP	*Homo sapiens* HepG2
ENCFF247KKW	ENCFF046IYE	Xiang-Dong Fu	SNRNP70	*Homo sapiens* HepG2
ENCFF490QZJ	ENCFF774URE	Xiang-Dong Fu	SRSF9	*Homo sapiens* HepG2
ENCFF641AUO	ENCFF883UWI	Xiang-Dong Fu	U2AF2	*Homo sapiens* HepG2
ENCFF033GIO	ENCFF739WOA	Xiang-Dong Fu	RBFOX2	*Homo sapiens* HepG2
ENCFF146SND	ENCFF560SVR	Xiang-Dong Fu	HNRNPL	*Homo sapiens* HepG2
ENCFF267HPS	ENCFF824EFD	Xiang-Dong Fu	AGO1	*Homo sapiens* HepG2
ENCFF728YOO	ENCFF542MVV	Xiang-Dong Fu	PRPF4	*Homo sapiens* HepG2
ENCFF707ALH	ENCFF954VXM	Xiang-Dong Fu	FIP1L1	*Homo sapiens* HepG2
ENCFF178TOV	ENCFF479DBP	Xiang-Dong Fu	PCBP2	*Homo sapiens* HepG2
ENCFF001IIN	ENCFF094XRA	Barbara Wold	USF1	*Homo sapiens* Myocyte
ENCFF001IIK	ENCFF302FXR	Barbara Wold	TCF3	*Homo sapiens* Myocyte
ENCFF001IHL	ENCFF770ANH	Barbara Wold	MYOD1	*Homo sapiens* Myocyte
ENCFF001IFF	ENCFF147NVK	Barbara Wold	MAX	*Homo sapiens* Myocyte
ENCFF001IIB	ENCFF380GQR	Barbara Wold	SRF	*Homo sapiens* Myocyte
ENCFF001IDK	ENCFF586VMG	Barbara Wold	E2F4	*Homo sapiens* Myocyte
ENCFF001IHG	ENCFF204AZL	Barbara Wold	MYOD1	*Homo sapiens* Myocyte
ENCFF001IIE	ENCFF727WYC	Barbara Wold	TCF12	*Homo sapiens* Myocyte
ENCFF001IFQ	ENCFF570IDO	Barbara Wold	REST	*Homo sapiens* Myocyte
ENCFF001IGZ	ENCFF259LKI	Barbara Wold	MYOG	*Homo sapiens* Myocyte

Table S2: ENCODE identifiers for ChIP-seq datasets and their controls

ChIP-seq	Control	Lab	Transcription Factor	Cell Line
ENCFF263MBV	ENCFF227IZS	Michael Snyder	TARDBP	*Homo sapiens* K562
ENCFF860IRS	ENCFF910IKB	Michael Snyder	TARDBP	*Homo sapiens* K562
ENCFF141XAC	ENCFF227IZS	Michael Snyder	SKIL	*Homo sapiens* K562
ENCFF995IZW	ENCFF910IKB	Michael Snyder	SKIL	*Homo sapiens* K562
ENCFF487SUP	ENCFF910IKB	Michael Snyder	SP1	*Homo sapiens* K562
ENCFF758LME	ENCFF227IZS	Michael Snyder	SP1	*Homo sapiens* K562
ENCFF254IUH	ENCFF227IZS	Michael Snyder	MTA2	*Homo sapiens* K562
ENCFF167NVK	ENCFF910IKB	Michael Snyder	MTA2	*Homo sapiens* K562
ENCFF294UTM	ENCFF227IZS	Michael Snyder	SMARCE1	*Homo sapiens* K562
ENCFF387BDW	ENCFF910IKB	Michael Snyder	SMARCE1	*Homo sapiens* K562
ENCFF619XNM	ENCFF910IKB	Michael Snyder	ATF7	*Homo sapiens* K562
ENCFF518PQY	ENCFF227IZS	Michael Snyder	ATF7	*Homo sapiens* K562
ENCFF024NZQ	ENCFF910IKB	Michael Snyder	RAD51	*Homo sapiens* K562
ENCFF168TBA	ENCFF227IZS	Michael Snyder	RAD51	*Homo sapiens* K562
ENCFF682OCC	ENCFF227IZS	Michael Snyder	MIER1	*Homo sapiens* K562
ENCFF639CLN	ENCFF910IKB	Michael Snyder	MIER1	*Homo sapiens* K562
ENCFF149LTH	ENCFF910IKB	Michael Snyder	ZBTB40	*Homo sapiens* K562
ENCFF873BRB	ENCFF227IZS	Michael Snyder	ZBTB40	*Homo sapiens* K562
ENCFF717OYW	ENCFF910IKB	Michael Snyder	ZEB2	*Homo sapiens* K562
ENCFF500YYZ	ENCFF227IZS	Michael Snyder	ZEB2	*Homo sapiens* K562
ENCFF073MBT	ENCFF214UMU	Tim Reddy	CEBPB	*Homo sapiens* A549
ENCFF280ZFT	ENCFF773DUX	Tim Reddy	CEBPB	*Homo sapiens* A549
ENCFF074CYV	ENCFF271MPT	Tim Reddy	EP300	*Homo sapiens* A549
ENCFF864XTU	ENCFF403EBP	Tim Reddy	EP300	*Homo sapiens* A549
ENCFF796UCB	ENCFF503AJR	Tim Reddy	BCL3	*Homo sapiens* A549
ENCFF098QVB	ENCFF831MPX	Tim Reddy	BCL3	*Homo sapiens* A549
ENCFF179XAQ	ENCFF171YYX	Tim Reddy	JUNB	*Homo sapiens* A549
ENCFF330XFU	ENCFF631DES	Tim Reddy	JUNB	*Homo sapiens* A549
ENCFF217RBI	ENCFF634ULC	Tim Reddy	CEBPB	*Homo sapiens* A549
ENCFF791DRP	ENCFF632UPH	Tim Reddy	CEBPB	*Homo sapiens* A549
ENCFF347MNU	ENCFF455UAB	Tim Reddy	CEBPB	*Homo sapiens* A549
ENCFF417GPF	ENCFF081TBO	Tim Reddy	CEBPB	*Homo sapiens* A549
ENCFF599JTK	ENCFF653HKQ	Tim Reddy	JUNB	*Homo sapiens* A549
ENCFF389OFH	ENCFF097CSC	Tim Reddy	JUNB	*Homo sapiens* A549
ENCFF476XBN	ENCFF193ABY	Tim Reddy	JUN	*Homo sapiens* A549
ENCFF761UEZ	ENCFF222ACA	Tim Reddy	JUN	*Homo sapiens* A549
ENCFF504YVD	ENCFF368OTV	Tim Reddy	HES2	*Homo sapiens* A549
ENCFF595EIS	ENCFF634ULC	Tim Reddy	HES2	*Homo sapiens* A549
ENCFF181HLP	ENCFF813FWF	Tim Reddy	NR3C1	*Homo sapiens* A549
ENCFF870WJP	ENCFF159VBZ	Tim Reddy	NR3C1	*Homo sapiens* A549

ChIP-seq	Control	Lab	Transcription Factor	Cell Line
ENCFF575GRT	ENCFF562HPN	Richard Myers	TAF1	*Homo sapiens* GM12878
ENCFF177XDM	ENCFF100EIH	Richard Myers	TAF1	*Homo sapiens* GM12878
ENCFF725DLZ	ENCFF438FFV	Richard Myers	EP300	*Homo sapiens* GM12878
ENCFF238NNG	ENCFF562HPN	Richard Myers	EP300	*Homo sapiens* GM12878
ENCFF399QIP	ENCFF100EIH	Richard Myers	BATF	*Homo sapiens* GM12878
ENCFF802IZN	ENCFF438FFV	Richard Myers	BATF	*Homo sapiens* GM12878
ENCFF579PRC	ENCFF754WTG	Richard Myers	RUNX3	*Homo sapiens* GM12878
ENCFF884LEJ	ENCFF966AVZ	Richard Myers	RUNX3	*Homo sapiens* GM12878
ENCFF791EPM	ENCFF562HPN	Richard Myers	PBX3	*Homo sapiens* GM12878
ENCFF845MYC	ENCFF100EIH	Richard Myers	PBX3	*Homo sapiens* GM12878
ENCFF963FTZ	ENCFF423BBH	Richard Myers	TARDBP	*Homo sapiens* GM12878
ENCFF179SRP	ENCFF747NOF	Richard Myers	TARDBP	*Homo sapiens* GM12878
ENCFF240MQI	ENCFF100EIH	Richard Myers	IRF4	*Homo sapiens* GM12878
ENCFF888PAI	ENCFF438FFV	Richard Myers	IRF4	*Homo sapiens* GM12878
ENCFF894EID	ENCFF430ZCF	Richard Myers	REST	*Homo sapiens* GM12878
ENCFF569QEN	ENCFF100EIH	Richard Myers	REST	*Homo sapiens* GM12878
ENCFF207QTV	ENCFF754WTG	Richard Myers	NFATC1	*Homo sapiens* GM12878
ENCFF983YCI	ENCFF966AVZ	Richard Myers	NFATC1	*Homo sapiens* GM12878
ENCFF731ZNW	ENCFF862QZT	Richard Myers	SRF	*Homo sapiens* GM12878
ENCFF263NOT	ENCFF289ONG	Richard Myers	SRF	*Homo sapiens* GM12878
ENCFF013XEG	ENCFF060VZM	Xiang-Dong Fu	TARDBP	*Homo sapiens* HepG2
ENCFF040PMB	ENCFF809MGU	Xiang-Dong Fu	TARDBP	*Homo sapiens* HepG2
ENCFF247KKW	ENCFF253UKR	Xiang-Dong Fu	SNRNP70	*Homo sapiens* HepG2
ENCFF046IYE	ENCFF279QEN	Xiang-Dong Fu	SNRNP70	*Homo sapiens* HepG2
ENCFF490QZJ	ENCFF629YZR	Xiang-Dong Fu	SRSF9	*Homo sapiens* HepG2
ENCFF774URE	ENCFF563YRA	Xiang-Dong Fu	SRSF9	*Homo sapiens* HepG2
ENCFF641AUO	ENCFF253UKR	Xiang-Dong Fu	U2AF2	*Homo sapiens* HepG2
ENCFF883UWI	ENCFF279QEN	Xiang-Dong Fu	U2AF2	*Homo sapiens* HepG2
ENCFF033GIO	ENCFF874GUL	Xiang-Dong Fu	RBFOX2	*Homo sapiens* HepG2
ENCFF739WOA	ENCFF719EDQ	Xiang-Dong Fu	RBFOX2	*Homo sapiens* HepG2
ENCFF146SND	ENCFF253UKR	Xiang-Dong Fu	HNRNPL	*Homo sapiens* HepG2
ENCFF560SVR	ENCFF279QEN	Xiang-Dong Fu	HNRNPL	*Homo sapiens* HepG2
ENCFF267HPS	ENCFF874GUL	Xiang-Dong Fu	AGO1	*Homo sapiens* HepG2
ENCFF824EFD	ENCFF719EDQ	Xiang-Dong Fu	AGO1	*Homo sapiens* HepG2
ENCFF728YOO	ENCFF450GNQ	Xiang-Dong Fu	PRPF4	*Homo sapiens* HepG2
ENCFF542MVV	ENCFF901YVZ	Xiang-Dong Fu	PRPF4	*Homo sapiens* HepG2
ENCFF707ALH	ENCFF707WXP	Xiang-Dong Fu	FIP1L1	*Homo sapiens* HepG2
ENCFF954VXM	ENCFF322OEH	Xiang-Dong Fu	FIP1L1	*Homo sapiens* HepG2
ENCFF178TOV	ENCFF450GNQ	Xiang-Dong Fu	PCBP2	*Homo sapiens* HepG2
ENCFF479DBP	ENCFF901YVZ	Xiang-Dong Fu	PCBP2	*Homo sapiens* HepG2

ChIP-seq	Control	Lab	Transcription Factor	Cell Line
ENCFF001IIN	ENCFF001IER	Barbara Wold	USF1	*Homo sapiens* Myocyte
ENCFF094XRA	ENCFF001IES	Barbara Wold	USF1	*Homo sapiens* Myocyte
ENCFF001IIK	ENCFF001IEN	Barbara Wold	TCF3	*Homo sapiens* Myocyte
ENCFF302FXR	ENCFF258NUS	Barbara Wold	TCF3	*Homo sapiens* Myocyte
ENCFF001IHL	ENCFF001IEN	Barbara Wold	MYOD1	*Homo sapiens* Myocyte
ENCFF770ANH	ENCFF258NUS	Barbara Wold	MYOD1	*Homo sapiens* Myocyte
ENCFF001IFF	ENCFF993HAY	Barbara Wold	MAX	*Homo sapiens* Myocyte
ENCFF147NVK	ENCFF290FPB	Barbara Wold	MAX	*Homo sapiens* Myocyte
ENCFF001IIB	ENCFF001IDR	Barbara Wold	SRF	*Homo sapiens* Myocyte
ENCFF380GQR	ENCFF122QLV	Barbara Wold	SRF	*Homo sapiens* Myocyte
ENCFF001IDK	ENCFF001IER	Barbara Wold	E2F4	*Homo sapiens* Myocyte
ENCFF586VMG	ENCFF993HAY	Barbara Wold	E2F4	*Homo sapiens* Myocyte
ENCFF001IHG	ENCFF001IDR	Barbara Wold	MYOD1	*Homo sapiens* Myocyte
ENCFF204AZL	ENCFF122QLV	Barbara Wold	MYOD1	*Homo sapiens* Myocyte
ENCFF001IIE	ENCFF001IES	Barbara Wold	TCF12	*Homo sapiens* Myocyte
ENCFF727WYC	ENCFF290FPB	Barbara Wold	TCF12	*Homo sapiens* Myocyte
ENCFF001IFQ	ENCFF001IEN	Barbara Wold	REST	*Homo sapiens* Myocyte
ENCFF570IDO	ENCFF258NUS	Barbara Wold	REST	*Homo sapiens* Myocyte
ENCFF001IGZ	ENCFF993HAY	Barbara Wold	MYOG	*Homo sapiens* Myocyte
ENCFF259LKI	ENCFF290FPB	Barbara Wold	MYOG	*Homo sapiens* Myocyte

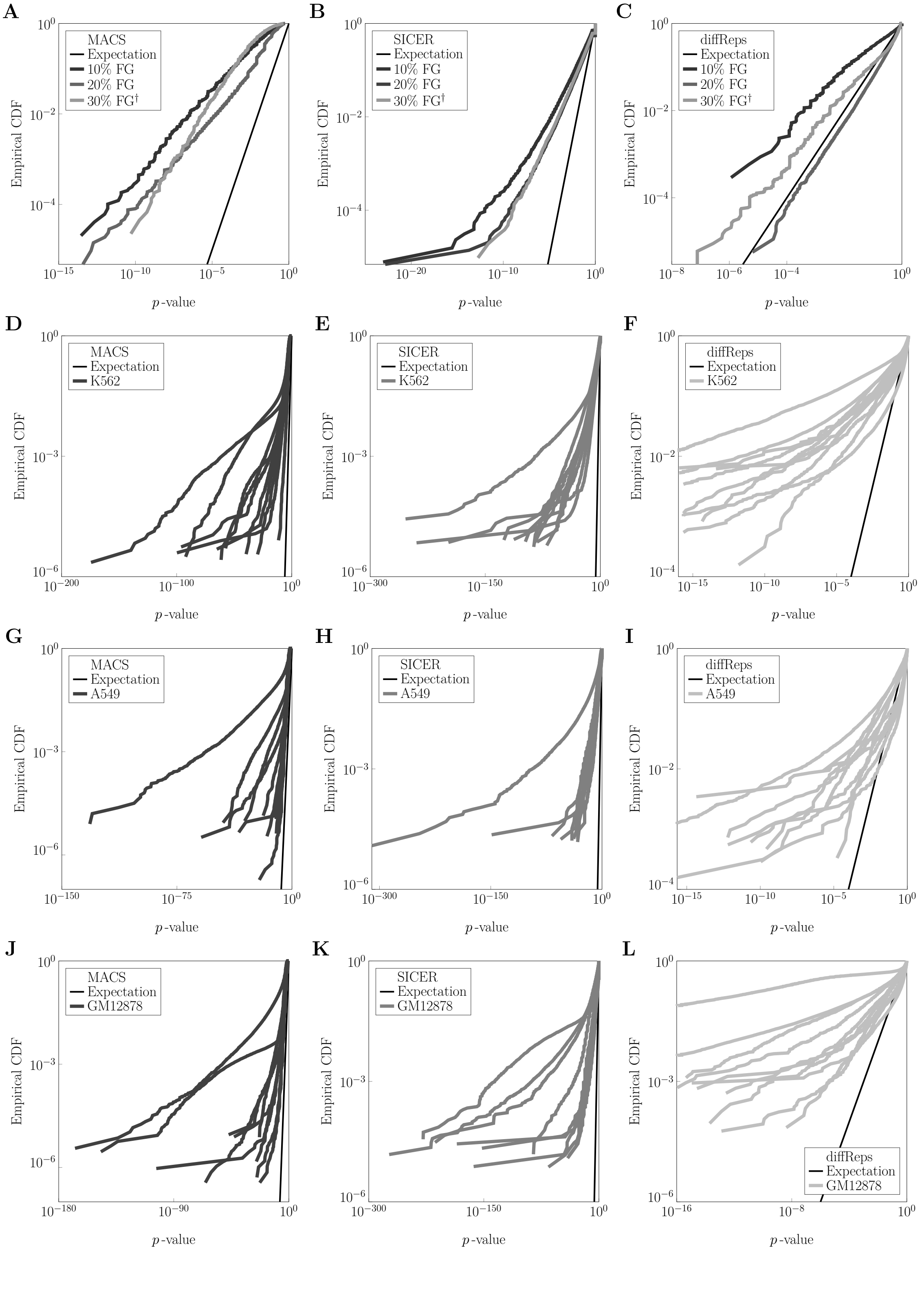
A
MACS
Expectation
10% FG
20% FG
30% FG†
Empirical CDF
p-value
B
SICER
Expectation
10% FG
20% FG
30% FG†
Empirical CDF
p-value
C
diffReps
Expectation
10% FG
20% FG
30% FG†
Empirical CDF
p-value
D
MACS
Expectation
K562
E
SICER
Expectation
K562
F
diffReps
Expectation
K562
G
MACS
Expectation
A549
H
SICER
Expectation
A549
I
diffReps
Expectation
A549
J
MACS
Expectation
GM12878
K
SICER
Expectation
GM12878
L
diffReps
Expectation
GM12878

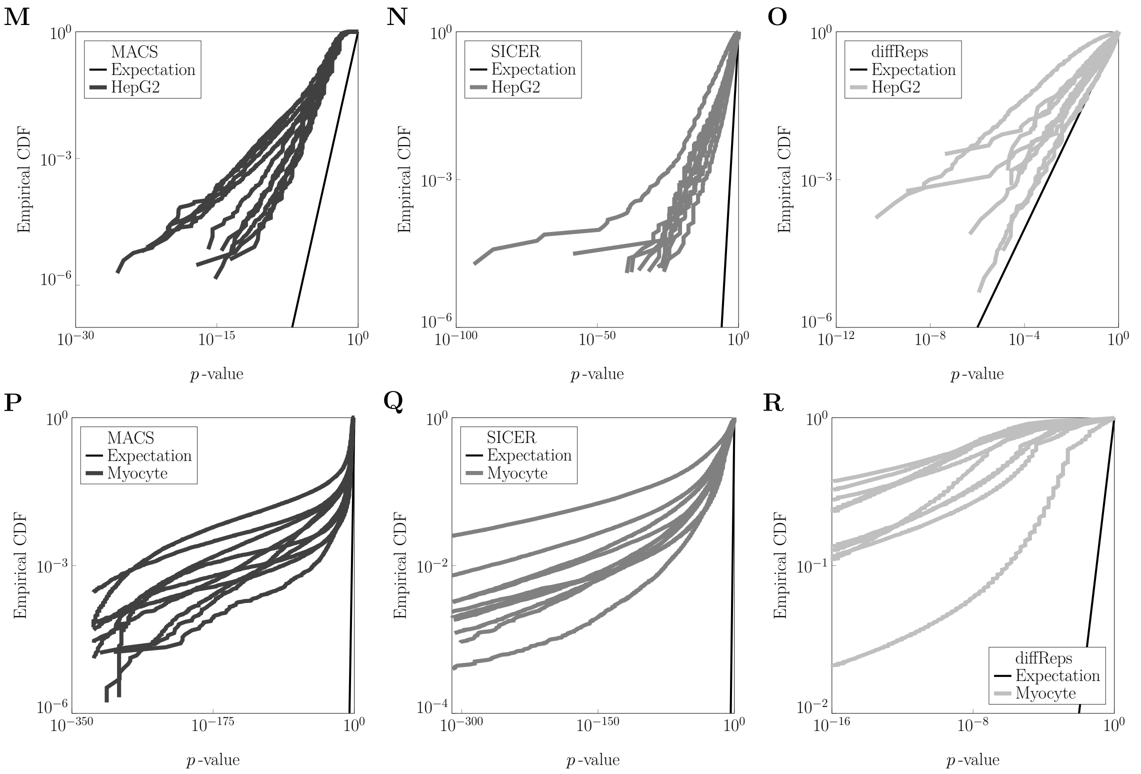

Figure S1: Empirical CDF for peak raw p-values for MACS (A), SICER (B), and diffReps (C) when applied to different types of simulated null hypothesis data: 500bp peaks with 10% of total reads, 500bp peaks with 20% of total reads, and 4kbp peaks with 30% of total reads. Empirical CDFs for peak raw p-values for MACS (D, G, J, M, P), SICER (E, H, K, N, Q), and diffReps (F, I, L, O, R) when applied to ENCODE data, where one ChIP-seq replicate is used as control for another ChIP-seq replicate.

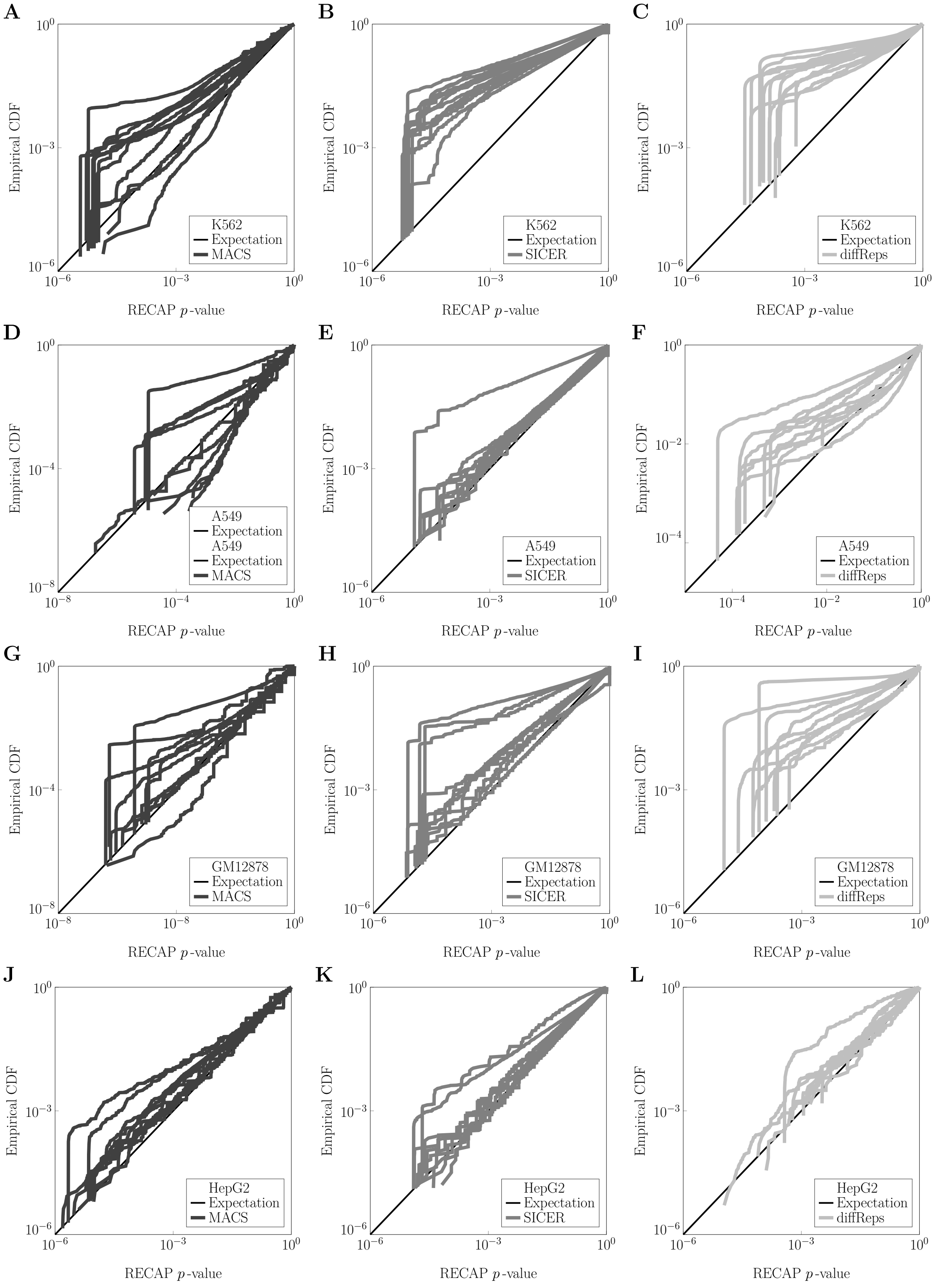

A
B
C
D
E
F
G
H
I
J
K
L
Empirical CDF
RECAP p-value
K562
Expectation
MACS
K562
Expectation
SICER
K562
Expectation
diffReps
A549
Expectation
A549
Expectation
MACS
A549
Expectation
SICER
A549
Expectation
diffReps
GM12878
Expectation
MACS
GM12878
Expectation
SICER
GM12878
Expectation
diffReps
HepG2
Expectation
MACS
HepG2
Expectation
SICER
HepG2
Expectation
diffReps

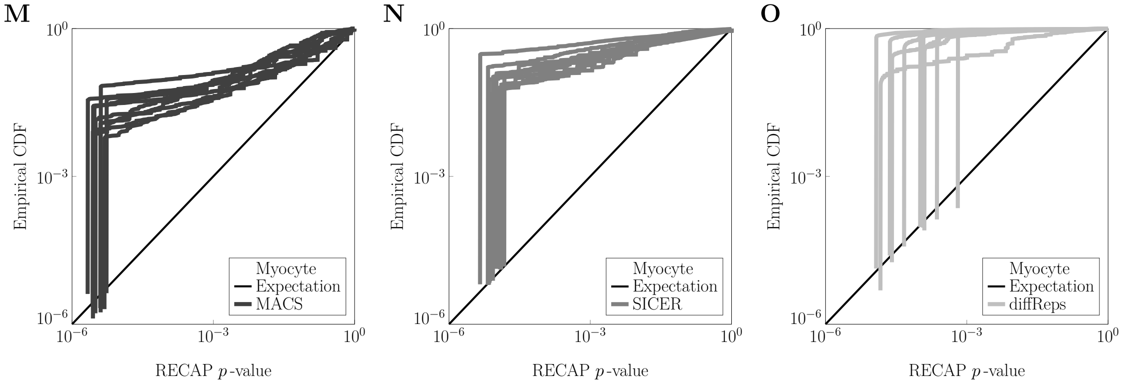

Figure S2: Empirical CDFs for peak recalibrated p-values for MACS (A, D, G, J, M), SICER (B, E, H, K, N) and diffReps (C, F, I, L, O) when applied to 10 replicate pairs of ENCODE ChIP-seq data.

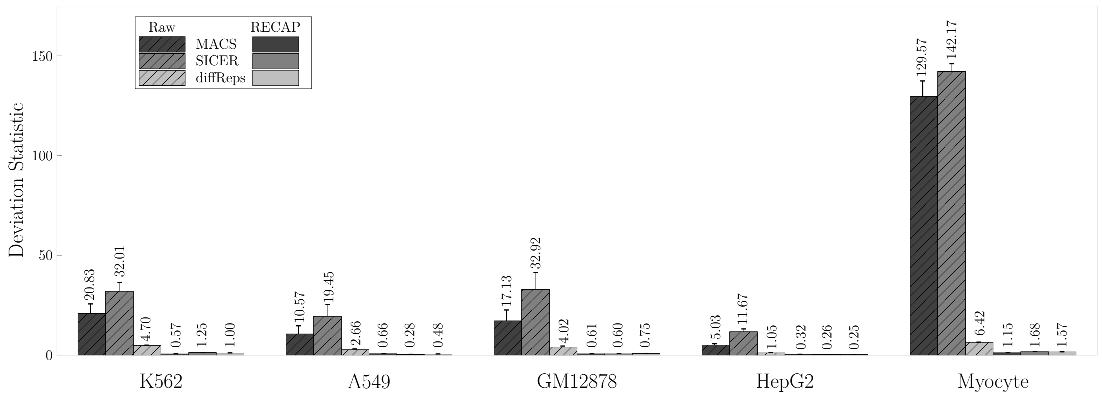

Figure S3: Reductions in deviation statistic for the RECAP recalibrated p-values for 10 replicate pairs of datasets within 5 cell lines.

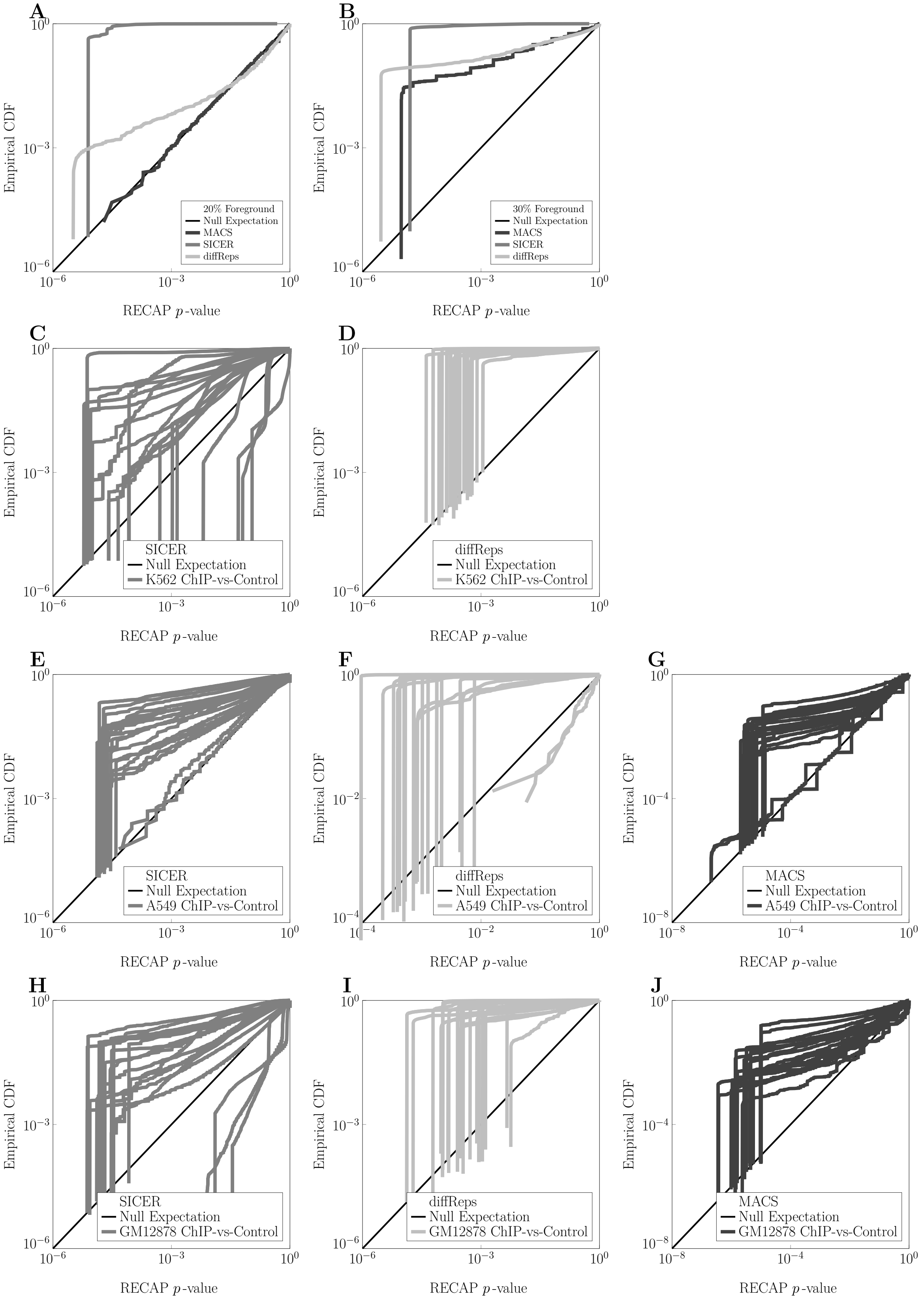
A
Empirical CDF
RECAP p-value
20% Foreground
Null Expectation
MACS
SICER
diffReps
B
Empirical CDF
RECAP p-value
30% Foreground
Null Expectation
MACS
SICER
diffReps
C
Empirical CDF
RECAP p-value
SICER
Null Expectation
K562 ChIP-vs-Control
D
Empirical CDF
RECAP p-value
diffReps
Null Expectation
K562 ChIP-vs-Control
E
Empirical CDF
RECAP p-value
SICER
Null Expectation
A549 ChIP-vs-Control
F
Empirical CDF
RECAP p-value
diffReps
Null Expectation
A549 ChIP-vs-Control
G
Empirical CDF
RECAP p-value
MACS
Null Expectation
A549 ChIP-vs-Control
H
Empirical CDF
RECAP p-value
SICER
Null Expectation
GM12878 ChIP-vs-Control
I
Empirical CDF
RECAP p-value
diffReps
Null Expectation
GM12878 ChIP-vs-Control
J
Empirical CDF
RECAP p-value
MACS
Null Expectation
GM12878 ChIP-vs-Control

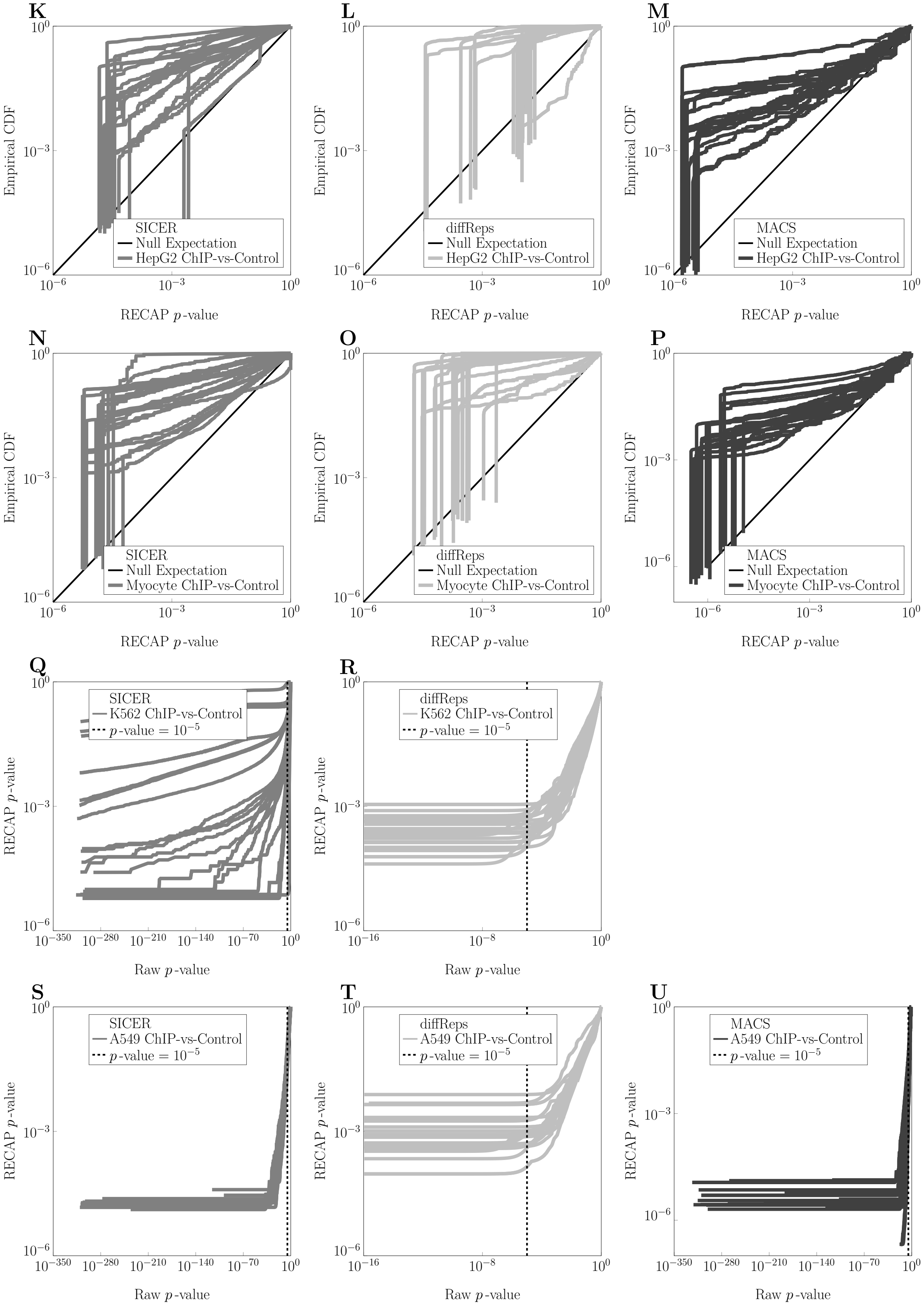
K
Empirical CDF
RECAP p-value
SICER
Null Expectation
HepG2 ChIP-vs-Control
L
diffReps
Null Expectation
HepG2 ChIP-vs-Control
M
MACS
Null Expectation
HepG2 ChIP-vs-Control
N
SICER
Null Expectation
Myocyte ChIP-vs-Control
O
diffReps
Null Expectation
Myocyte ChIP-vs-Control
P
MACS
Null Expectation
Myocyte ChIP-vs-Control
Q
SICER
K562 ChIP-vs-Control
p-value = 10^{-5}
Raw p-value
R
diffReps
K562 ChIP-vs-Control
p-value = 10^{-5}
S
SICER
A549 ChIP-vs-Control
p-value = 10^{-5}
T
diffReps
A549 ChIP-vs-Control
p-value = 10^{-5}
U
MACS
A549 ChIP-vs-Control
p-value = 10^{-5}

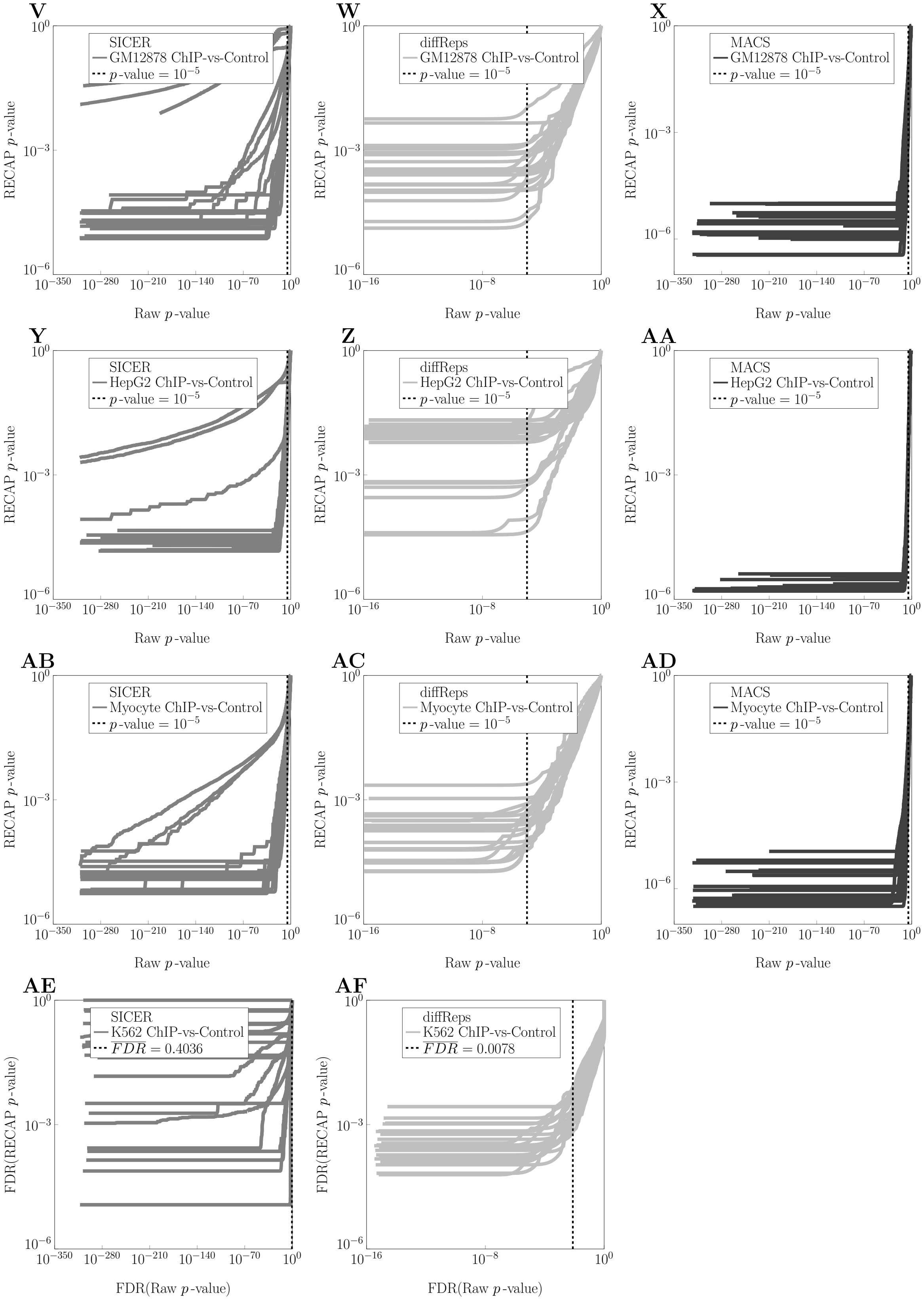

V
SICER
GM12878 ChIP-vs-Control
p-value = 10^-5
RECAP p-value
Raw p-value
W
diffReps
GM12878 ChIP-vs-Control
p-value = 10^-5
RECAP p-value
Raw p-value
X
MACS
GM12878 ChIP-vs-Control
p-value = 10^-5
RECAP p-value
Raw p-value
Y
SICER
HepG2 ChIP-vs-Control
p-value = 10^-5
RECAP p-value
Raw p-value
Z
diffReps
HepG2 ChIP-vs-Control
p-value = 10^-5
RECAP p-value
Raw p-value
AA
MACS
HepG2 ChIP-vs-Control
p-value = 10^-5
RECAP p-value
Raw p-value
AB
SICER
Myocyte ChIP-vs-Control
p-value = 10^-5
RECAP p-value
Raw p-value
AC
diffReps
Myocyte ChIP-vs-Control
p-value = 10^-5
RECAP p-value
Raw p-value
AD
MACS
Myocyte ChIP-vs-Control
p-value = 10^-5
RECAP p-value
Raw p-value
AE
SICER
K562 ChIP-vs-Control
FDR = 0.4036
FDR(RECAP p-value)
FDR(Raw p-value)
AF
diffReps
K562 ChIP-vs-Control
FDR = 0.0078
FDR(RECAP p-value)
FDR(Raw p-value)

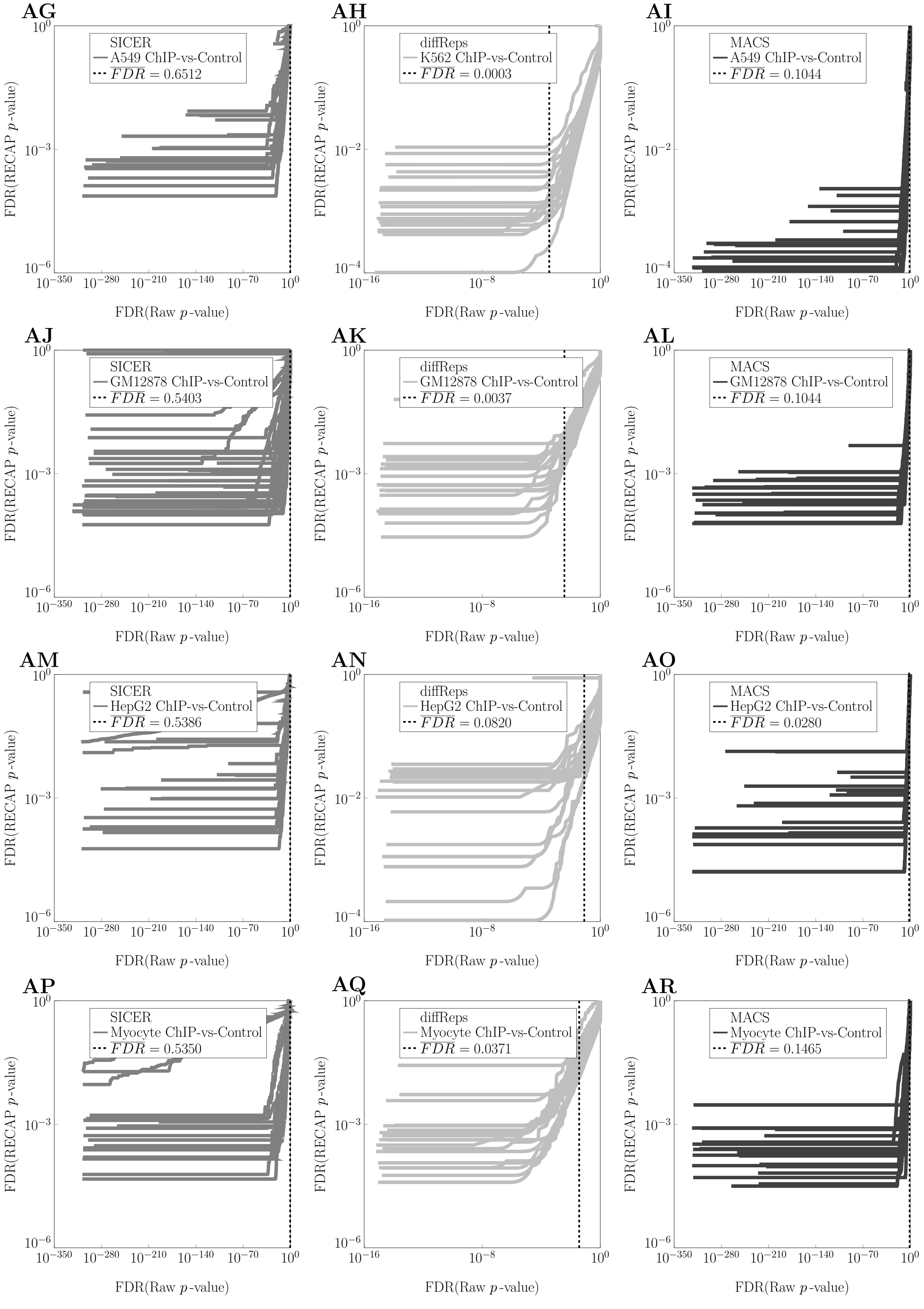

AG
SICER
A549 ChIP-vs-Control
$\overline{FDR} = 0.6512$
AH
diffReps
K562 ChIP-vs-Control
$\overline{FDR} = 0.0003$
AI
MACS
A549 ChIP-vs-Control
$\overline{FDR} = 0.1044$
AJ
SICER
GM12878 ChIP-vs-Control
$\overline{FDR} = 0.5403$
AK
diffReps
GM12878 ChIP-vs-Control
$\overline{FDR} = 0.0037$
AL
MACS
GM12878 ChIP-vs-Control
$\overline{FDR} = 0.1044$
AM
SICER
HepG2 ChIP-vs-Control
$\overline{FDR} = 0.5386$
AN
diffReps
HepG2 ChIP-vs-Control
$\overline{FDR} = 0.0820$
AO
MACS
HepG2 ChIP-vs-Control
$\overline{FDR} = 0.0280$
AP
SICER
Myocyte ChIP-vs-Control
$\overline{FDR} = 0.5350$
AQ
diffReps
Myocyte ChIP-vs-Control
$\overline{FDR} = 0.0371$
AR
MACS
Myocyte ChIP-vs-Control
$\overline{FDR} = 0.1465$
FDR(RECAP p-value)
FDR(Raw p-value)
10^{0}
10^{-2}
10^{-3}
10^{-4}
10^{-6}
10^{-350}
10^{-280}
10^{-210}
10^{-140}
10^{-70}
10^{-16}
10^{-8}

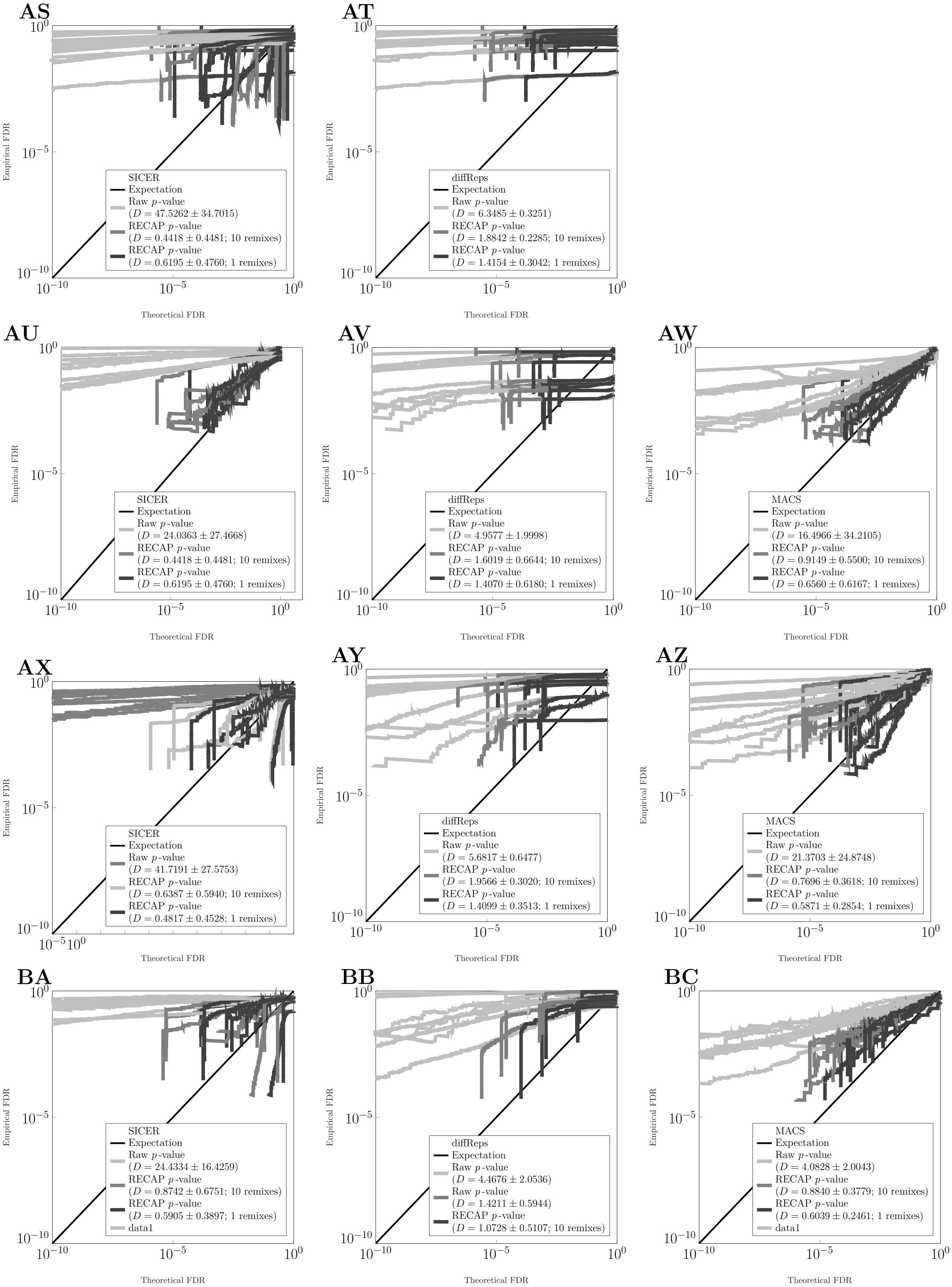
AS
Empirical FDR
Theoretical FDR
SICER
Expectation
Raw p-value
(D = 47.5262 ± 34.7015)
RECAP p-value
(D = 0.4418 ± 0.4481; 10 remixes)
RECAP p-value
(D = 0.6195 ± 0.4760; 1 remixes)
AT
Empirical FDR
Theoretical FDR
diffReps
Expectation
Raw p-value
(D = 6.3485 ± 0.3251)
RECAP p-value
(D = 1.8842 ± 0.2285; 10 remixes)
RECAP p-value
(D = 1.4154 ± 0.3042; 1 remixes)
AU
Empirical FDR
Theoretical FDR
SICER
Expectation
Raw p-value
(D = 24.0363 ± 27.4668)
RECAP p-value
(D = 0.4418 ± 0.4481; 10 remixes)
RECAP p-value
(D = 0.6195 ± 0.4760; 1 remixes)
AV
Empirical FDR
Theoretical FDR
diffReps
Expectation
Raw p-value
(D = 4.9577 ± 1.9998)
RECAP p-value
(D = 1.6019 ± 0.6644; 10 remixes)
RECAP p-value
(D = 1.4070 ± 0.6180; 1 remixes)
AW
Empirical FDR
Theoretical FDR
MACS
Expectation
Raw p-value
(D = 16.4966 ± 34.2105)
RECAP p-value
(D = 0.9149 ± 0.5500; 10 remixes)
RECAP p-value
(D = 0.6560 ± 0.6167; 1 remixes)
AX
Empirical FDR
Theoretical FDR
SICER
Expectation
Raw p-value
(D = 41.7191 ± 27.5753)
RECAP p-value
(D = 0.6387 ± 0.5940; 10 remixes)
RECAP p-value
(D = 0.4817 ± 0.4528; 1 remixes)
AY
Empirical FDR
Theoretical FDR
diffReps
Expectation
Raw p-value
(D = 5.6817 ± 0.6477)
RECAP p-value
(D = 1.9566 ± 0.3020; 10 remixes)
RECAP p-value
(D = 1.4099 ± 0.3513; 1 remixes)
AZ
Empirical FDR
Theoretical FDR
MACS
Expectation
Raw p-value
(D = 21.3703 ± 24.8748)
RECAP p-value
(D = 0.7696 ± 0.3618; 10 remixes)
RECAP p-value
(D = 0.5871 ± 0.2854; 1 remixes)
BA
Empirical FDR
Theoretical FDR
SICER
Expectation
Raw p-value
(D = 24.4334 ± 16.4259)
RECAP p-value
(D = 0.8742 ± 0.6751; 10 remixes)
RECAP p-value
(D = 0.5905 ± 0.3897; 1 remixes)
data1
BB
Empirical FDR
Theoretical FDR
diffReps
Expectation
Raw p-value
(D = 4.4676 ± 2.0536)
Raw p-value
(D = 1.4211 ± 0.5944)
RECAP p-value
(D = 1.0728 ± 0.5107; 10 remixes)
BC
Empirical FDR
Theoretical FDR
MACS
Expectation
Raw p-value
(D = 4.0828 ± 2.0043)
RECAP p-value
(D = 0.8840 ± 0.3779; 10 remixes)
RECAP p-value
(D = 0.6039 ± 0.2461; 1 remixes)
data1

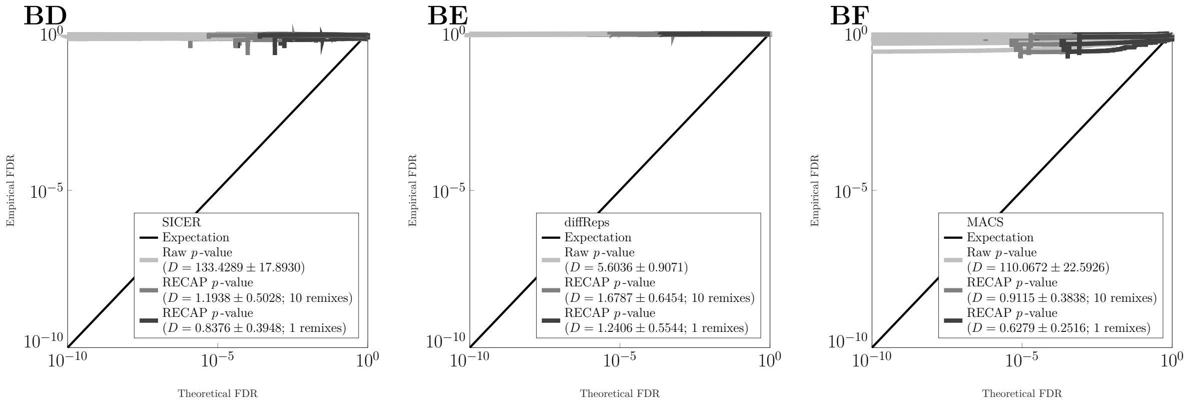

Figure S4: Empirical CDFs, p-values and theoretical and empirical FDRs for SICER, diffReps, and MACS for ENCODE ChIP-seq versus control peak calling.

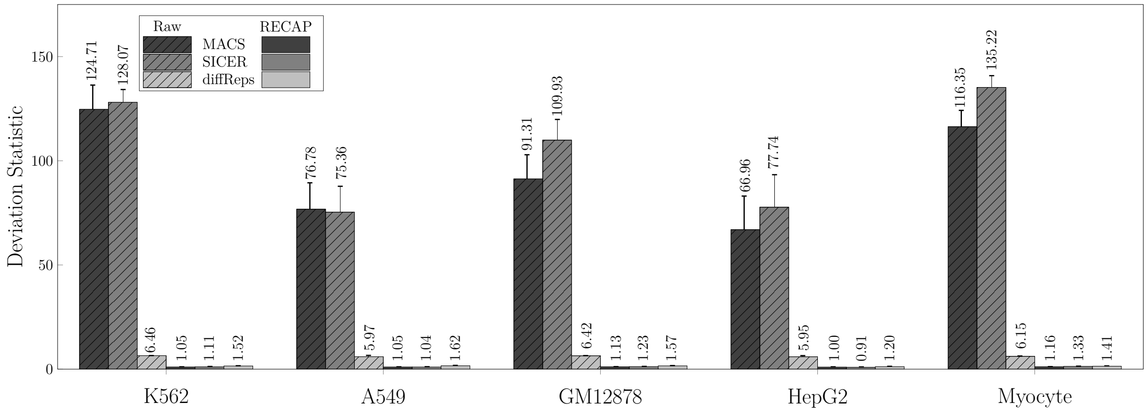

Figure S5: Reductions in deviation statistic for the RECAP recalibrated p-values for 10 pairs of ENCODE ChIP-seq versus control peak calling within 5 cell lines.
